# Reductive Stress in Inflammation-Associated Diseases and the Pro-Oxidant Effect of Antioxidant Agents

**DOI:** 10.3390/ijms18102098

**Published:** 2017-10-05

**Authors:** Israel Pérez-Torres, Verónica Guarner-Lans, María Esther Rubio-Ruiz

**Affiliations:** 1 Department of Pathology, Instituto Nacional de Cardiología “Ignacio Chávez”, Juan Badiano 1, Sección XVI, Tlalpan, Mexico City 14080, Mexico; 2 Department of Physiology, Instituto Nacional de Cardiología “Ignacio Chávez”, Juan Badiano 1, Sección XVI, Tlalpan, Mexico City 14080, Mexico; veronica.guarner@cardiologia.org.mx (V.G.-L.); esther.rubio@cardiologia.org.mx (M.E.R.-R.)

**Keywords:** reductive stress, antioxidants, reducing equivalents, inflammation, pro-oxidants

## Abstract

Reductive stress (RS) is the counterpart oxidative stress (OS), and can occur in response to conditions that shift the redox balance of important biological redox couples, such as the NAD^+^/NADH, NADP^+^/NADPH, and GSH/GSSG, to a more reducing state. Overexpression of antioxidant enzymatic systems leads to excess reducing equivalents that can deplete reactive oxidative species, driving the cells to RS. A feedback regulation is established in which chronic RS induces OS, which in turn, stimulates again RS. Excess reducing equivalents may regulate cellular signaling pathways, modify transcriptional activity, induce alterations in the formation of disulfide bonds in proteins, reduce mitochondrial function, decrease cellular metabolism, and thus, contribute to the development of some diseases in which NF-κB, a redox-sensitive transcription factor, participates. Here, we described the diseases in which an inflammatory condition is associated to RS, and where delayed folding, disordered transport, failed oxidation, and aggregation are found. Some of these diseases are aggregation protein cardiomyopathy, hypertrophic cardiomyopathy, muscular dystrophy, pulmonary hypertension, rheumatoid arthritis, Alzheimer’s disease, and metabolic syndrome, among others. Moreover, chronic consumption of antioxidant supplements, such as vitamins and/or flavonoids, may have pro-oxidant effects that may alter the redox cellular equilibrium and contribute to RS, even diminishing life expectancy.

## 1. Introduction

**Figure 1:**
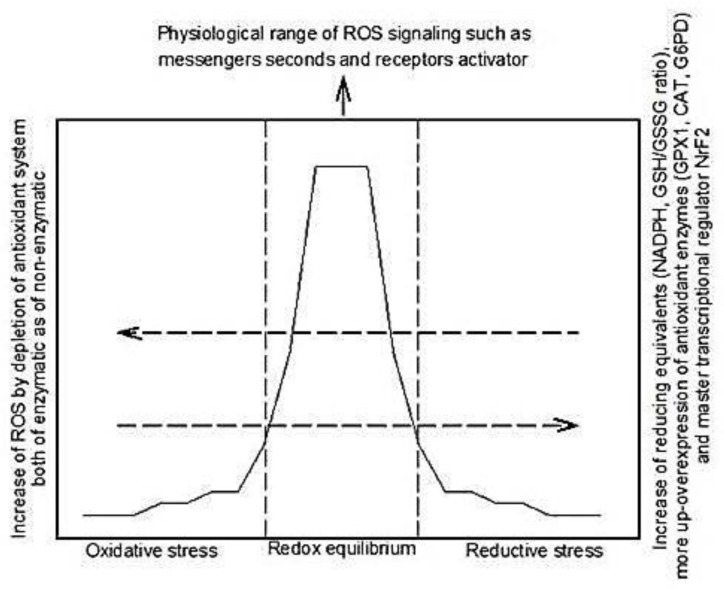
The redox equilibrium is essential for cellular homeostasis; moderate reactive oxygen species (ROS) production leads to their effects as second messengers. However, ROS overproduction and/or depletion or the antioxidant enzymatic and non-enzymatic systems may lead to oxidative stress. Excess reducing equivalents such as glutathione reduced (GSH)/glutathione oxidized (GSSG) ratio and nicotinamide adenine dinucleotide reduced (NADPH) can depleted all ROS driving to reductive stress by overexpression of antioxidant enzymatic system. Moreover, chronic reductive stress may induce an oxidative stress and stimulated reductive stress by a feedback regulation. Nevertheless, this process it is not yet clearly understood. Adapted from Lubos et al., 2011 [2] and Brewer et al., 2011 [3].

Redox equilibrium is essential for cellular homeostasis. It moderates reactive oxidative species (ROS) production, leading to their effects as second messengers. However, ROS overproduction and/or depletion of the enzymatic and non-enzymatic antioxidant systems may lead to oxidative stress (OS) and its consequences. On another hand, the excess of reducing equivalents that result from an elevation in the GSH/GSSG and/or NAD/NADH^+^ ratio or overexpression of antioxidant enzymatic systems can deplete all ROS driving the cells to RS (Figure 1). Reductive stress (RS) is defined as an abnormal increase of reducing equivalents in the presence of intact systems for oxidation and reduction [1]. Excess reducing equivalents diminish cell growth responses, induce alterations in the formation of disulfide bonds in proteins, reduce mitochondrial function and decrease cellular metabolism. It might contribute to the development of some diseases that are closely associated to inflammatory conditions, such as aggregation protein cardiomyopathy, hypertrophic cardiomyopathy, muscular dystrophy, pulmonary hypertension, rheumatoid arthritis, cancer, Alzheimer’s disease, and metabolic syndrome, among others. In this review, we cover the knowledge on RS, in which there are still many questions to be answered; RS participation in different diseases, which involve inflammatory conditions; and the adverse effects of antioxidant agents, and their impact on living beings.

## 2. Reactive Oxidative Species and Antioxidant Systems

When a balance between ROS production and the enzymatic and non-enzymatic antioxidant systems is present, the organism is found in redox equilibrium, which is essential for many biological processes. However, when there is an increase in ROS or reduced activity of one or two antioxidant systems, the result is OS [1,4]. The redox equilibrium is necessary for cellular homeostasis and a moderate ROS production leads to the effects caused by second messengers of oxygen species, such as nitric oxide (NO), nitrogen monoxide (•NO), and hydrogen peroxide (H_2_O_2_) [4]. NO and •NO act as messenger molecules that contribute to vasodilation, proliferation, and promote or counteract programmed and spontaneous cell apoptosis and necrosis [5]. H_2_O_2_ modulates the transduction of signals by reversible oxidation of proteins at cysteine, which has redox activity. It also oxidizes thiols in tyrosine kinase phosphatases [6]. Two-electron enzymatic reductions of molecular oxygen by oxidases, such as xanthine oxidase, can also produce superoxide (O_2_^−^) [7]. The dismutation of O_2_^−^ by superoxide dismutase (SOD) induces the formation of H_2_O_2_. This reaction may also happen spontaneously. In mammalian cells, H_2_O_2_ may activate at least 40 gene products [8,9]. Likewise, a reduced level of H_2_O_2_ may promote apoptosis. In cell systems, when the antioxidant enzyme catalase (CAT) is overexpressed in the cytoplasmic or mitochondrial compartments, there is potentiated apoptosis [10]. In contrast, inhibition of endogenous CAT promotes cell survival [11]. Additional studies have tied the CAT-induced decrease in H_2_O_2_ with diminished activation of NF-κB survival pathways. These pathways are necessary to counteract apoptotic signaling [12,13]. In contrast, when few ROS are produced or the antioxidant systems are upregulated, RS and its consequences appear (Figure 1).

## 3. Reductive Stress

RS is a condition where a relative shortage of ROS, compared with reducing equivalents in the form of redox couples NAD/NADH^+^, NADPH/NADP^+^, and GSH/GSSG, is present [14,15]. RS has a deleterious effect in lower eukaryotes and in cells from many species, including mammalian organisms [16]. NADH is an electron carrier whose excess may lead to pathogenic mitochondrial oxidation and breakdown of in vivo and in vitro mitochondrial homeostasis, and to misfolding of proteins in the endoplasmic reticulum (ER) [17]. In addition, chronic RS can induce OS, which stimulates again RS by a feedback regulation. For example, during RS, when electron acceptors are expected to be mostly reduced, some redox proteins can donate electrons to O_2_ instead, thus increasing ROS production [18]. However, a high level of reducing equivalents also enhances ROS scavenging systems, involving redox couples such as the NAD/NADH^+^, NADPH/NADP^+^, and glutathione reduce (GSH)/glutathione oxidized (GSSG) ratio [18,19], resulting in a net H_2_O_2_ spillover from mitochondria that favors RS [19].

On the other hand, the term mitochondrial homeostasis refers to how low doses of mitochondrial ROS produced by the respiratory electron transport chain (RETC) can activate the biogenesis and the antioxidant capacity, in order to counteract OS and to re-establish homeostasis [1]. Besides, energy production requires intracellular redox homeostasis that is coordinated and regulated by a mechanism linked to networks of key signal transduction and mitochondrial oxidative phosphorylation. Each of the individual organelles has a different redox requirement, mainly of GSH/GSSG ratio [17].

Mitochondrial ROS and their depletion by RS play an essential and necessary role in the correct folding of proteins and in the formation of disulfide bonds, which determine the normal structure and function of many proteins [19]. When the mitochondrial oxidant production is inhibited, there is an important decrease in the levels of cellular disulfide bonds in many cells [20]. RS leads to the loss of disulfide bond formation and induces the unfolded protein response of the ER endoplasmic reticulum (UPR^ER^). The recuperation of the correct folding of proteins is necessary to regain proteostasis in this compartment [21]. It has been reported that H_2_O_2_ accumulation during RS attenuated the UPR^ER^ amplitude by altering translation, without any discernible effect on transcription in *Saccharomyces cerevisiae* [22]. In yeast with RS, some proteins showed delayed folding, disordered transport and failed oxidation, and were finally aggregated [15].

## 4. Participation of Different Molecules in Reductive Stress

**Figure 2:**
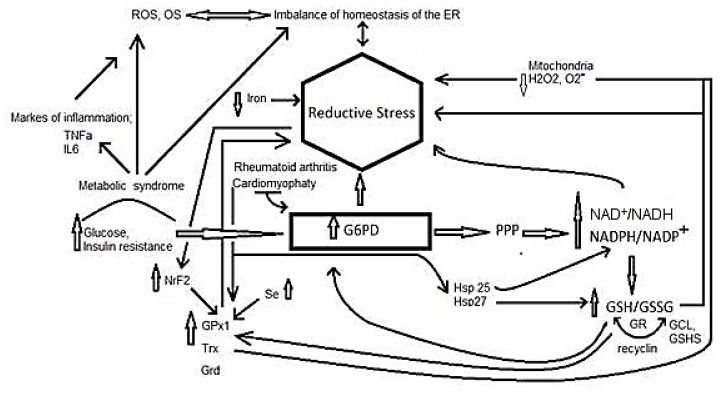
Participation of several agents such as the reducing equivalents, antioxidant enzymes and pathologies in reductive stress. Abbreviations: G6PD = glucose 6 phosphate dehydrogenase, NAD = nicotinamide adenine dinucleotide, NAD^+^ = nicotinamide adenine dinucleotide oxidized, NADH = nicotinamide adenine dinucleotide reduced, NADPH = nicotinamide adenine dinucleotide phosphate reduced, GSH = glutathione, GSSG = glutathione disulfide, PPP = pentose phosphate pathway, γ-glutamyl-cysteine synthase, GSHS = glutathione synthetase, GPx = Glutathione peroxidase, Trx = thioredoxin, Grd = glutaredoxin, TNFα = tumor necrosis factor alpha, NrF2 = erythroid related factor 2, IL6 = interleukin 6, ROS = reactive oxidative species, OS = oxidative stress, ER = endoplasmic reticulum, Se = selenium, Hsp = heat shock protein, GR = glutathione reductase.

Mechanisms for the generation of RS and participation of diverse agents, such as the reducing equivalents, antioxidants enzymes, and pathologies, are summarized in Figure 2.

### 4.1. Nicotinamide Adenine Dinucleotide oxidized/Nicotinamide Adenine Dinucleotide Reduced Ratio

The coenzyme nicotinamide adenine dinucleotide (NAD) is a ubiquitous biological redox cofactor that is formed by two nucleotides that are linked by their phosphate groups. One nucleotide contains an adenine base, and the other nicotinamide. It is present in two forms, an oxidized NAD^+^, and reduced form NADH. NAD^+^ is a singly charged anion (charge of −1), while NADH is a doubly charged anion [23].

The ratio between the oxidized and reduced forms participates in redox reactions, carrying electrons from one reaction to another. NADH can be used as a reducing agent to donate electrons [4]. Although the main function of this ratio is the electron transfer reactions, it is also used in other cellular processes, such as being a substrate of enzymes that add or remove chemical groups from proteins, and in posttranslational modifications. The ratio participates in many functions, such as energy metabolism, mitochondrial functions, calcium homeostasis, antioxidation/generation of OS, gene expression, immunological functions, aging, and cell death. NADH acts as an antioxidant and its excess can induce RS [23].

NAD^+^ can be synthesized from simple building blocks, from tryptophan or aspartic acid, or it can be taken up from the vitamin niacin. NAD^+^ can also be transformed into nicotinamide adenine dinucleotide phosphate (NADP), whose chemistry is similar to that of NAD, but has different roles in metabolism [23].

Furthermore, overproduction of NADH or lack of NAD^+^ can induce the accumulation of NADH [24]. Overproduction of NADH induces an electron pressure upon mitochondrial complex I, which responds within its capacity, to oxidize more NADH to NAD^+^. This leads to an increase in electron leakage that decreases oxygen to yield O_2_^−^. These free radicals, in turn, enhance OS. Due to a high level of reducing equivalents, such as NADH, an oxidative condition appears [25], and it achieves the transition to RS by the polyol pathway. This pathway converts NADPH to NADH, leading to a redox imbalance between NADH and NAD^+^ [26]. This condition could be linked to metabolic syndrome (MS) and diabetes.

Nicotinamide adenine dinucleotide phosphate (NADP^+^) differs from NAD^+^ in the presence of an additional phosphate group on the ribose ring. NADPH is the reduced form of NADP^+^. The NADP^+^ is a cofactor used in the synthesis of lipids and nucleic acids and other anabolic reactions, which require NADPH as a reducing agent. An important ROS producing system is the NADPH oxidase family (NOX) in cardiac myocytes and many other cell types, including neurons [27]. This system can be activated by RS. When the dominant negative NOX4 expression is elevated in mice, it abolishes the NOX function, producing an importantly reducing state (high GSH/GSSG, low NADP^+^/NADPH), and it directly activates nuclear erythroid-related factor 2 (Nrf2) [28].

### 4.2. Reduced Glutathione/Disulfide Glutathione Ratio

GSH is a tripeptide formed by glutamate, cysteine, and glycine, having a low molecular weight that has been widely used as an indicator of the cellular redox state, and has been implicated in several pathologies. It is synthetized by γ-glutamyl-cysteine synthetase (GCL), GSH synthetase, and regenerated by glutathione reductase (GR) [11]. GSH is the endogenous intracellular antioxidant found in a higher concentration within cells that acts against ROS and electrophiles, and is one of the main mechanisms for the antioxidant defense. Approximately 15% is bound to proteins, and the rest of it is found in a free form [29]. GSH inactivates O_2_^−^ and OH^−^ radicals, and transforms vitamins E and C into their active forms [30].

Reduced plasma and cellular levels of GSH signify the presence of OS [31]. When ROS are present, GSH is oxidized to GSSG [32]. GSSG can also accumulate inside the cell and react with the sulfhydryl groups of proteins to produce GSH-disulfide proteins, which have longer half-lives, and as a consequence, reduce the amount of poorly folded protein [13]. The enzymes involved in the biosynthesis and generation of GSH, including GCL, GSH synthase, GR, and glucose-6-phosphate dehydrogenase (G6PD), are derived from antioxidant genes regulated by NrF2 [33]. GCL catalyzes the rate-limiting step in GSH synthesis by regulating the formation of γ-glutamyl-cysteine from glutamine and cysteine. Increases in its expression, lead to higher GSH concentrations that could be used to stop ROS in OS conditions. When the cell is unable to maintain the GSH intracellular concentration, irreversible cell damage happens, thus playing a central role in the antioxidant defenses [34]. GSH is a molecule that consumes reducing equivalents and has been implicated in several pathologies.

GSH excess could decrease the basal ROS and contribute to RS [34,35]. In the cytosol, the GSH/GSSG ratio ranges from 30:1 to 100:1, with a redox potential of −290 mV. In the ER, the GSH/GSSG ratio ranges between 1:1 to 3:1, having a redox potential (a tendency to acquire electrons) that ranges from −170 to −185 mV [32], and in the mitochondria, the range of the GSH/GSSG ratio falls within 20:1 to 40:1, with a redox potential of 1250 to −280 mV [35].

The availability of GSH for mitochondrial peroxidases is elevated by its mitochondrial import via the 2-oxoglutarate and dicarboxylate carriers, thereby affecting H_2_O_2_ levels. Furthermore, GSH biosynthesis increases the mitochondrial pool, modifying the RETC that elevates O_2_^−^ production. Increased MnSOD enhances H_2_O_2_ generation [17].

### 4.3. Glutathione Peroxidase 1 Isoform

The glutathione peroxidase (GPx) isoform family consists of homologous enzymes that contain a selenium-cysteine. One of the most plentiful members of the GPxs family is GPx1. It is the main antioxidant enzyme preventing the accumulation of damaging intracellular H_2_O_2_. It uses GSH as a source of reducing equivalents [36]. The human GPx1 gene is localized in human chromosome 3p21 [37]. It is more effective than CAT in removing intracellular peroxides under many physiological conditions, and can reduce lipid hydrogen peroxides, and decrease lipid peroxidation (LPO) [38]. GPx1 may also act as a peroxynitrite reductase to modulate in vivo ONOO^−^ flux, since the studies suggest that lack of GPx1 enhances survival to ONOO^−^ [39]. However, GPx1 overexpression can occur because of substrate surplus [2].

Furthermore, ROS are required for keeping the formation of disulfide formation in the cells, and GPx1 overexpression can reduce formation of protein disulfide, a mechanism that depends on the oxidant generation of mitochondria, and mitochondrial uncoupling [40]. Excess GPx1 leads to a decrease of protein disulfides that is related to reduced signaling from growth factors and a decreased mitochondrial function, characterized by a lower mitochondrial potential and a reduced ATP generation [40].

### 4.4. Thiols

Low molecular weight thiols play an important role in redox-mediated processes in the cell. Thiol groups react with electrophiles and oxidants, and have high affinities for metals, rendering them adaptable to many biological roles. There is a delicate balance between the productive and the pathogenic reactions occurring among thiol groups [41]. Thiol oxidation and reduction in biological systems leads to the formation of various reversible and irreversible products that can be recovered through the action of cellular reductants, like GSH and thioredoxin (Trx). Among the products of Cys oxidation, sulfenic acids, *S*-nitrosothiols, and disulfides are of particular interest, given their roles in redox cycling and/or regulation of enzymes and transcription factors involved in cell signaling processes [41]. Indeed, Trx exerts immunomodulatory properties and pro-inflammatory effects by regulating NF-κB [42]. The Trx/peroxy-redoxin/methionine sulfoxide reductase pathway and the GSH/GPx/glutathione-*S*-transferase (GST)/glutaredoxin (Grd) are the primary redox regulatory systems for the control of the cellular redox environment. These systems contain the small heat-stable oxido reductases Trx, and Grd, which contain thiol groups in their active sites, formed by two cysteine residues [43]. They act as hydrogen donors for ribonucleotide reductase, and are necessary for many metabolic enzymes that have a disulfide bond in their catalytic site. Their roles include regulation of protein folding, decrease of dehydroascorbate, and the reparation of proteins altered by oxidative processes and sulfur metabolism [44].

The forms of Trx having an oxidized disulfide are reduced by NADPH and Trx reductase, while the forms of Grd are reduced by GSH, employing NADPH-donated electrons [44]. In the ER, the oxidative range of protein folding is 1:1 to 3:1, with respect to the ratio GSH/GSSG. Therefore, disulfide formation is dependent on the compartmentalization of oxidative chemistry. This prevents the exposition of the cell to non-specific oxidation events, in which the GSH/Grd and Trx systems are needed for redox homeostasis. The loss of Trx or Trx reductase leads to an imbalance in the GSH/GSSG ratio, and thus, the redox state that increases sensitivity to RS [15].

Trx maintains redox homeostasis in response to both oxidative and RS conditions, particularly, it is required for protection against RS through the exposition to dithiothreitol (DTT) in the yeast *Saccharomyces cerevisiae*. DTT is a small dithiol compound designed and employed as a potent reducing agent that can be tolerated by cells. Its reducing potential is responsible for its ability to autoxidize, and generates O_2_^−^ in oxygenated solutions [45]. In the yeast model, RS seems to be a consequence of elevated GSH levels, and results in a constitutively high proportion of unfolded proteins in the ER [15]. Increased concentrations of the GSH/GSSG ratio can also be produced by deletion of Trx reductase in yeast. This ratio reversibly regulates the Trx function through glutathionylation [17], which is defined as post-translational modification of a protein through a disulfide bond by reaction with GSH [46].

In addition, high levels of GSSG in the ER provide an oxidizing redox potential that drives protein disulfide formation, increases thiols that are toxic to eukaryotic cells, pushes the thiol redox potential of the ER to the reducing direction, and disrupts protein disulfide formation and protein folding [47]. In a similar manner, GPx, GR, and peroxy-redoxin/Trx/TrxR2 systems, can leak electrons to O_2_^−^, and generate a significant amount of ROS spillover when the supply of their natural electron acceptors is limited or electron transport to acceptors is inhibited, leading to RS. This provides new insights into how RS is generated by ROS production [18].

Moreover, persulfide species, such as cysteine persulfide (CysSSH), play important roles in the regulation of redox cell signaling, as part of the antioxidant response [48]. Indeed, these species can interact with GSH to form glutathione persulfide (GSSH) and/or transfer the sulfur group to Cys residues of different proteins, to produce its polysulfidation that can regulate the protein activity [49]. However, the physiological role of persulfide species remains poorly studied.

### 4.5. Iron

Iron is an essential cofactor for important biological activities and biochemical reactions, and iron metabolism constitutes redox-based machinery that is essential to metabolic requirements. Iron plays a critical role in the generation of O_2_^−^ through the Haber-Weiss-Fenton reactions. Under conditions of increased OS, this machinery becomes a potential threat, exacerbating the pro-oxidant condition. A decrease in intracellular iron content diminishes ROS generation, and may lead to RS by feedback regulation [50]. Low intracellular free iron downregulates ferritin, the protein that stores iron and releases it in a controlled fashion, and upregulates transferrin receptor 1 (TFR1) that is a carrier protein for transferrin needed for the import of iron into the cell [50]. Increased NADPH levels may also favor the Fe(II) state, aiding in the incorporation of iron into ferritin.

### 4.6. Selenium

Selenium (Se), an essential nutritional trace element, is considered a non-antioxidant system, and it is exclusively obtained from the diet, and is considered a metalloid of interest from the perspective of toxicologists and nutritionists [51]. Several biological functions in the human body depend on the balance of Se levels, and decreased or elevated levels can cause damaging effects. Se is very important for different Se-proteins; 25 Se-proteins are present in humans and 24 homologues have been found in rodents [52]. They participate in different physiological processes, such as chemoprevention, neurobiology, aging, immunity, anti-inflammatory activity, muscle metabolism, reproduction, and redox reactions [52]. Se is present in foods and dietary supplements in different chemical forms, such as Se-methionine, Se-cysteine, selenite, sodium selenite, and selenious acid [53]. The synthesis of Se proteins such as GPx isoforms is affected by levels of Se supplementation; however, exceeding and inadequate Se intake can produce damaging health effects and contribute to RS by upregulated Se-protein W (SelW) mRNA expression [54]. This enzyme belongs to a subfamily of Se-dependent proteins that includes SelV, SelT, and SelH forms, mixed disulfites with substrate proteins that bind to DNA in a redox-sensitive manner. SelT participates in mobilization of Ca^2+^ and metabolism of glucose, while SelM and Sel15 function as oxide-reductases in the ER lumen [52]. These Se-enzymes increase antioxidant capacity, and alter the inflammatory signaling pathways that modulate ROS by inhibiting the NF-κB cascade. However, NF-κB can increase the expression of antioxidant enzymes, leading to a diminished synthesis and release of interleukins and tumor necrosis factor alpha (TNF-α) [55].

Moreover, NF-κB and AP-1 can regulate the promoters of some antioxidant enzymes, besides regulating the expression of the enzymes involved in GSH synthesis. However, the most important factor in the antioxidant response is NrF2 [56].

### 4.7. Nuclear Erythroid-Related Factor 2

Redox-sensitive NrF2 is a leucine zipper protein that contributes to RS and acts as an important transcriptional regulator of several hundred cytoprotective and antioxidant genes [57]. When OS is present, NrF2 is separated from Keap-1, moves into the nucleus, and activates antioxidant enzyme gene expression. In conditions of RS, an alternative mechanism for Nrf2 target gene activation has been described; in this situation, high levels of reducing agents can lead to RS and elevated levels of the autophagy adaptor p62/SQSTM1, which is also linked to Keap-1, reducing NrF2 cytoplasmic sequestration, and allowing for NrF2 nuclear translocation and target gene activation This mechanism relies on the competition between Nrf2 and p62/SQSTM1, an autophagy cargo acceptor, for the binding of Keap-1 (its negative regulator), then, it is ubiquinated and degraded by the proteasome [3,58]. Additionally, when OS is present, activation of NrF2, after being dissociated and released from Keap1, results in its transfer to the nucleus, where it combines to cis-acting AREs or electrophile response elements, and leads to the transcription of several antioxidant and cytoprotective genes, such as GST, heme oxygenase-1, Trx, NQO1, and GLC [59].

## 5. Reductive Stress in Inflammation Related Diseases

Mechanisms of RS generation involved on the development of inflammation-associated diseases are summarized in Table 1.

### 5.1. Reductive Stress and Cardiac Health

The pathophysiology of heart diseases is complex and multifactorial, and several molecular pathways are involved. These molecular pathways may be interconnected, and some of them have been related to the increase GSH/GSSG ratio, i.e., the presence of RS. One of the pathways is the activation of inflammatory signaling pathways. Pro-inflammatory cytokines exert strong direct effects on cardiomyocytes, inducing apoptosis, depression of contractility, and down-regulation of sarcomeric proteins [77]. Another molecular pathway is the deficient expression of chaperones, protein quality control pathways and heat shock proteins (Hsp). Small Hsp are ubiquitously present in cells, protecting them from stress through their chaperone, anti-apoptotic, and anti-inflammatory activities in a variety of tissues, including the heart, brain, and immune system [78].

Rajasekaran first reported the presence of RS in mice expressing the human mutant αB-crystallin protein. Dominant mutations in genes that encode for chaperones, such as *CryAB* and Bag3, determine many inherited human disorders, including protein aggregation cardiomyopathy, skeletal muscle myopathy, and cataracts [79,60]. Mutations in the small molecular weight Hsp αB-crystallin (CryAB) or in desmin (an intermediate filament cytoskeletal protein that maintains muscle integrity and tolerance to stress) cause protein aggregation, skeletal myopathies, and cardiomyopathies, in which there is misfolding of proteins and the presence of large cytoplasmic aggregates.

A cardiomyopathy included in multisystem protein aggregation diseases is caused by an autosomal dominant mutation in the human αB-crystallin gene, inducing a R120G amino acid exchange. Despite the fact that the pathogenesis of the cardiomyopathy found in this mutant, hR120G CryAB, is poorly understood, the development of cardiac hypertrophy and heart failure from human hR120G CryAB, causes deregulation of GSH homeostasis that leads to RS in transgenic mice. However, transgenic mice overexpressing cardiac-specific hR120G CryAB recapitulate the cardiomyopathy characteristics present in humans, and these mice are under RS. The hearts with myopathy show an increased recycling of GSSG to GSH, which is due to the augmented expression and enzymatic activities of G6PD, GR, and GPx. In a mouse model of cardiomyopathy, there was enhanced activity of G6PD with increased production of NADPH and higher levels of GSH, resulting in protein aggregation [60,80]. Therefore, G6PD activity could be a target for the treatment of R120G CryAB cardiomyopathy and heart failure in humans [59].

In addition, the human mutant αB-crystallin protein further induced expression of Hsp, in particular, Hsp25, which participates in RS through increased levels of expression of cardiac NADPH, GSH, G6PD, CAT, and GPx1 isoform [61,62].

Overexpression of Hsp27 can induce RS and cardiomyopathy, in part by the up regulation of GPx1 expression [64]. In L929 cells, the overexpression of Hsp27 decreased the intracellular iron and carbonyl protein content [81,82]. In CCL39 cells, Hsp27 overexpression caused a decreased iron level [82]. Hsp27 may downregulate TFR1 mediated iron uptake via stabilization of the cortical actin cytoskeleton in CCL39 cells [82]. Hsp27 overexpression may lead to iron deficiency in myopathy hearts, but not in lungs and livers, with up regulation of GPx1 that decreases H_2_O_2_ concentration and leads to RS [64]. In another study with cardiac overexpression of Hsp27, there was RS with elevated GSH, GSH/GSSG ratio, GPx1, and decreased ROS levels, resulting in cardiac hypertrophy and dysfunction in a similar way to that of Hsp25 [64]. NrF2 activation is controversial, although there is evidence that NrF2 may improve cardiac pathology [83], however, it has also been associated with a variety of cardiac pathologies [84]. Its participation occurs in two stages: initially due to ROS generation, and later, due to Keap1 dysfunction through its sequestration into the mutant protein aggregates [85]. This results in sustained activation and nuclear translocation of NrF2, and leads to ceaseless transcriptional upregulation of antioxidant enzymes contributing to RS [63]. Under this condition, the reductive capacity of the cell, and/or the concentration of reducing equivalents with increased of GSH levels and NADPH, exceeds ROS production (feedback mechanism) [59].

However, NrF2 deficiency reduces aggregation of mutant proteins. This suggests that oxidative modification of intracellular proteins is an event needed for adequate ubiquitination and protein degradation, which decreases cardiomyopathy in RS [86]. NrF2 deficiency is associated with significant GSH depletion in vivo and in vitro, which in turn will prevent RS in the transgenic mice myocardium [87].

Furthermore, several studies have described the presence of chronic Se deficiency in patients suffering from a rapidly progressive cardiomyopathy or extensive fibrosis [88]. A high Se-status could have adverse cardio-metabolic effects of on cardiovascular diseases (CVD) [89]. Higher plasma Se levels were associated with increased total lipoproteins and low density lipoprotein (LDL), and the risk of dyslipidemia [90,91]. A potential explanation between high Se and high lipid levels is a shared enzyme, 3-hydroxy-3-methyglutaryl coenzyme A reductase that can act through the mevalonate pathway that affects both Se and lipids [91]. A cross-sectional study of 1859 participants aged 65 or older, from four rural regions in China, showed an association between high plasma Se levels and the risk of high-triglycerides (TG). Subjects carrying the APOEε4 have higher rates of high-total cholesterol (TC) and high-LDLC [91].

In the heart under perfusion conditions, the decrease in NOX function produces a reducing state. Inhibition of NOX causes an even more important damage by ischemia/reperfusion (I/R) [28]. The abolished NOX function prevents the accumulation of HIF1α, and consequently, impairs the switch of fatty acid to glucose utilization during I/R, and thus increases damage, causing more severe damage [92].

### 5.2. Reductive Stress and Pulmonary Hypertension

Pulmonary hypertension is a progressive and multifactorial disease characterized by vasoconstriction, vascular remodeling, and micro thrombotic events. In this pathology, inflammation plays an important role due to the accumulation of perivascular inflammatory cells (macrophages, dendritic cells, T and B lymphocytes, and mast cells) and because circulating pro inflammatory cytokines are increased [93].

The presence of RS related to hypoxia has been described in pulmonary vascular cells, and may participate in the pathogenesis of pulmonary hypertension. Hypoxia causes a 2-fold increase in intracellular 2-oxoglutarate (2OG) together with an increase in reduced 2-hydroxyglutarate (2HG). There are two enantiomers of 2HG; the d and l enantiomers, which have been associated with rare inborn errors of metabolism, resulting in increased urinary excretion of 2HG, linked to neurological deficits in children [94]. Both enantiomers inhibit 2OG-dependent deoxygenates which favor the response to mitochondrial RS caused by the respiratory chain, tricarboxylic acid cycle dysfunction. These perturbations increase mitochondrial NADH and provide the substrate for L2HG production and accumulation, which participate in the increase in RS [65].

### 5.3. Reductive Stress and Stent Stenosis

Risk factors for vascular remodeling, such as hypertension, endothelial dysfunction, diabetes and atherosclerotic plaque formation, inflammation and vascular injury during stent implantation, are associated with reductions in the enzyme GPx1 [95]. However, increases in this enzyme lead to RS by enhancing GSH/GSSG and NADPH/NADP^+^ ratios. RS, in turn, elevates *s*-glutathionylation of important proteins, a process which leads to vascular smooth muscle cell proliferation, migration, and survival, contributing to stent stenosis [66]. The expression and activity GPx1 are dependent on many factors, including diet levels of Se [52].

### 5.4. Reductive Stress and Neuro-Muscular Disorders

Neuromuscular diseases frequently involve chronic muscle inflammation that is accompanied by muscle weakness. Furthermore, inflammation damage may affect the arteries and blood vessels that run through the muscle. Some neuromuscular disorders are present at birth, while others manifest in childhood, and even during the adult stage [66]. These diseases can be due to genetic mutations, to abnormal immune responses, or to the effect of toxins or tumors [84].

Higher increases in the GSH/GSSG ratio elevate mitochondrial oxidation, and induce cytotoxicity in cultured cells and in models of muscular dystrophy [60]. The *Drosophila melanogaster* model was used to study the mutations in lamins identified in muscular dystrophy patients, showing that aggregation of cytoplasmic lamins are associated with elevated levels of GSH and NADPH, and with elevated p62/SQSTM1, and nuclear enrichment of NrF2, leading to RS [84]. These increases in the ROS production could cause a change in the intracellular GSH redox state to generate more reduced intracellular equivalents (high GSH/GSSG ratio). This demonstrates that while an initial stimulus might be oxidative in nature, the response of the cell can subsequently result in an overall more reducing cellular environment and lead to RS [96].

In skeletal muscle and in the muscle-derived C2C12 cell, the insecticide, piscicide, and pesticide, rotenone, led to a profound deposition of intracellular triacylglycerol accumulation via inhibition of the RETC complex I, and increased ratio of NAD^+^/NADH that was associated with accumulation of lipids and RS that impaired muscle contraction [97].

There are neurotoxic effects of Se. Se induced a decrease in locomotion, generalized muscular flaccidity and a catalepsy-like state. There was also a decrease in respiratory and heart rates that were followed by respiratory death and cardiac arrest [98]. Also, the neurotoxic effects inducible by Se include an increase of dopamine levels in the central nervous system [67], a reduction of the global antioxidant status, sulfhydryl groups, and LPO [99]. In rat sciatic nerve fibers, it induced neuromuscular blockade, tetanic spasm, alteration of nerve fiber action potentials, and nerve membrane depolarization [68]. There was a significant elevation of Se and iron in motor neuron disease that was associated with an increase in the activity of GPx that could to lead to RS [69]. In addition, the over expression of Se-antioxidant enzymes like the GPx is regulated by transcription factors such as NF-κB, activator protein-1 (AP-1) and NrF2 [100]. The expression of many genes that participate in inflammation, embryonic development, oncogenesis, and apoptosis is regulated by NF-κB and AP-1. Moreover, these transcription factors appear to be activated simultaneously by the same stimuli and control the same cell signaling pathways [101].

### 5.5. Parkinson’s Disease

Neurodegenerative diseases are characterized by the death of neurons in different regions of the nervous system, followed by a deterioration of the affected parts. Although the mechanism that unleashes and leads the chronic process in these pathologies remains unknown, inflammation is a common factor that is accompanied by an increased production of protein aggregates and alterations in the neurotransmitter concentrations [102]. Parkinson’s disease has been attributed to the interference with the electron-transfer from iron-sulfur centers to ubiquinone in the complex I of RETC caused by the increment of NAD^+^/NADH [70].

### 5.6. Reductive Stress in Insulin Resistance Associated with Metabolic Syndrome

Chronic over nutrition with high sucrose creates chronic hyperglycemia that can induce MS. The induced MS includes obesity, hypertension, dyslipidemia, insulin resistance (IR), hyperinsulinemia [103], and insulin secretion impairment [104]. Under hyperglycemic conditions, more glucose flows through the glycolytic pathways that produce pyruvate and acetyl-CoA, leading to more NADH production. More glucose can also stimulate the glyceraldehyde-3-phosphate dehydrogenase that leads to more NADH through glycolysis and the Krebs cycle. In addition, under hyperglycemic conditions, the polyol pathway utilizes more than 30% of the body glucose, which significantly contributes to RS [105]. Moreover, iNOS also uses NADPH as a cofactor, contributing to hypertension in MS. Therefore, RS followed by OS could act as an important process of glucotoxicity when chronic hyperglycemic conditions are present. It would induce RS, which is linked to the inhibition of insulin release by pancreatic β-cells [24]. Previous studies have shown that a decreased activity of the RETC complex I is associated with obesity, type II diabetes and lipid accumulation in skeletal muscle [106]. Furthermore, in a GPx1 overexpressing male mice model that is characterized by IR, hyperglycemia, hyperinsulinemia, increased fat deposits and plasma leptin, and diminished insulin sensitivity. GPx-1 activity overexpression may interfere with the insulin function by over-quenching intracellular ROS required for insulin sensitizing [73]. H_2_O_2_ can undoubtedly modulate the insulin induced phosphorylation of the β-subunit of the insulin receptor [107], and protein kinase B (PkB, also known as Akt) [108]. Insulin stimulation generates a burst of H_2_O_2_ in hepatoma and adipose cells that is associated with a reversible oxidative inhibition of overall cellular protein tyrosine phosphatase activity. Therefore, the regulation of reversible tyrosine phosphorylation in the insulin signaling cascade is essential for keeping the normal activity of protein tyrosine phosphatase and insulin sensitivity [109]. Insulin signaling through the Akt phosphorylation of Ser^473^ requires of the presence of normal or minimal levels of intracellular ROS or H_2_O_2_ to be sensitized [58,73]. The extinction of the intracellular H_2_O_2_ blast after insulin stimulation is accelerated by the overexpression of GPx-1, resulting in more activity of protein tyrosine phosphatase, and reduced phosphorylation of the insulin receptor [106,73]. In GPx-1 overexpressing mice, increases of GPx-1 activity, ranging from 31 to 300%, were related to obesity and IR, and phosphorylation of Akt was reduced in response to insulin [110].

There is also an association between CAT and GPx-1. CAT overexpression prevented IR in muscle cells chronically exposed to fatty acids by improving mitochondrial function, and consequently, glucose and fatty acid metabolism through a decrease of H_2_O_2_ [110,111]. These findings with excess CAT and GPx-1 suggest that apoptosis might increase as a result of disrupted oxidant signaling, thus increasing RS [2]. The modification of Bax/Bcl-2 ratio environment can be listed as one of the molecular targets affected by the increased expression of GPx-1 [112]. In transgenic mice, the overexpression of the SOD and GPx-1 alter functions, including an increased expression of immediate early genes and proteins, and also results in dysfunction in thermoregulation and the appearance of a thermo sensitive phenotype [113,114].

The association between a decrease of H_2_O_2_ by GPx-1 overexpression leads to RS. Many actions of insulin participate as effector mechanisms of pro-inflammatory processes involved in the development of cardiovascular disorders. IR is defined as a loss of sensitivity to the hormone by the cells, and reduced or absent metabolic responses that promote glucose homeostasis [115]. IR and its consequent hyperinsulinemia are one of the first signs of MS [116]. Recent evidence indicates that inflammatory pathways are causally involved in IR. In particular, inflammation can directly impair the insulin signaling pathway mediated by serine phosphorylation of the insulin receptor substrates (IRS), and/or indirectly, via induction of transcription of pro-inflammatory mediators [116,117]. Insulin actions are exerted through activation of two transduction signaling pathways. Metabolic actions, as well as vasodilator endothelial actions, such as oxide nitric (ON) production, are mediated through fosfatidyl-inositol-3 kinase pathway (PI3K). Mitogenic actions, growth, and cellular differentiation, are mediated by MAPK and particularly, the C-Jun N-terminal kinases (JNK) subfamily, which controls the pro-inflammatory cytokine-expression of TNF-α and IL-6 [118]. IR increases TNF-α and IL-6 concentrations, and these cytokines reduce insulin action by (a) activating JNK-1 kinase, which phosphorylates IRS-1; (b) inducing the activation of NF-κB [119]. TNF-α action is blocked in isolated cells, as well as in whole animals, and insulin sensitivity is restored [115]. A sensitive marker that predicts the risk of developing CVD is C-reactive protein (hs-CRP), which is an acute inflammatory molecule formed in the liver by IL-6 and TNF-α [26].

Moreover, loss of ER homeostasis or unusually high UPR^ER^ induced by RS is closely associated with multiple complex disorders, including MS, type II diabetes, and CVD [22].

### 5.7. Reductive Stress and Rheumatoid Arthritis

CD4 T-cells in patients with rheumatoid arthritis promote synovitis, autoantibody formation, facilitate osteoclast differentiation, and impose endothelial dysfunction and pro-inflammatory effector functions. These T-cells, like malignant cells, depend on oxidative glucose metabolism coupled with mitochondrial oxidative phosphorylation to efficiently generate ATP [120]. However, to replicate from a single cell into thousands of copies, they need a carbon source and the reducing power of NADH, in addition to ATP [74]. Naïve rheumatoid arthritis (RA) T-cells have a defect in the glycolytic flux due to up regulation of G6PD. The excess G6PD shunts glucoses into the pentose phosphate pathway (PPP), resulting in an increase and accumulation of NADPH that leads to consumption of all ROS, resulting in RS. The insufficient oxidative signaling prevents the activation of the cell cycle kinase ATM and allows RA T-cells to bypass the G2/M cell cycle checkpoint, thus creating an inflammation-prone T-cell pool [74]. Several metabolic interventions, such as the use of several drugs, are able to rebalance glucose utilization away from the PPP and towards glycolytic breakdown, easing RS and preventing hyper proliferation and incorrect differentiation of RA T-cells.

### 5.8. Reductive Stress and Renal Diseases

The products of the prototypical glucose regulated (*grp*) genes: *grp94* and *grp78* play important roles as chaperones during protein folding and processing in the ER [76], and are also linked to inflammatory conditions, such renal disease. These genes are members of the gene battery that is responsive to RS, while the *hsp* genes respond to OS [121,122]. Thiol reductions are also cytotoxic and increase expression of *grp* genes. Agents that interfere with ER protein folding include thiols that activate *grp78* transcription [75]. In LLC-PK1 renal epithelial cells, DTT treatment induces *grp78* gene expression and *gadd153* gene transcription. In addition, in human embryonic kidney cells *N*-acetyl-l-cysteine treatment led to 3- to 4-fold increase of GSH. This increased the level of mitochondrial oxidation, and drove to RS that could later on lead to oxidative stress [14]. RS associated to hypoxia causes the L2HG enantiomer accumulation in renal cell carcinoma of children. Cell lines with RETC defects and D2HG have been identified as the product of cancer-associated mutant enzyme cytosolic isocitrate dehydrogenase-1 [65].

### 5.9. Reductive Stress in Infectious Diseases

Pathogens that produce diseases have also been related to RS. In *Mycobacterium tuberculosis* (Mtb), DTT exposure leads to thiol RS that derives in the formation of an adherent biofilm in Mtb cultures. Metabolically active and drug-tolerant bacteria are found in these biofilms [123]. Bacteria develop an envelope where periplasmic proteins are unfolded in response to thiol RS. The presence of this envelope leads to the upregulation of a specific transcriptional response [124].

### 5.10. Reductive Stress in Alzheimre’s Diseases

In an Alzheimer’s disease (AD) model, the APP/PS1 transgenic mice, RS occurs at a young age and before the onset of the disease [71]. RS in this model is characterized by increased G6PD and GSH that contribute to damage of the mitochondrial membrane sulfhydryl groups, which are rendered susceptible by the depletion of H_2_O_2_ [72]. Young healthy individuals at risk of AD also suffer from RS, in which there is overexpression of antioxidant enzymes before the onset of the disease. Therefore, it is a paradox why this hyperresponse of the antioxidant defenses drives subjects to RS collapses at some point during the development of the disease, leading to OS, which finally contributes to the development of dementia [125].

## 6. Situations Inducing Non-Pathological Reductive Stress: Hypoxia and Exercise

The metabolic adaptation to hypoxia is critical for the survival, remodeling, and proliferation of cells. Hypoxia causes respiratory chain and tricarboxylic acid cycle dysfunction, and these alterations increase mitochondrial NADH and provide the substrate for reduced hydroxyglutarate production and accumulation, which participates in the increase in RS [65]. 

A study showed that exercise-induced RS in young men that performed a knee extensor session performing isokinetic eccentric exercise [126]. However, reports on this topic are scarce in the literature. Depending on the type, intensity and duration of the exercise, physical complexion and genetic background, the subjects exposed to exercise can be driven, or not, to RS. Exhaustive exercise can increase GPx, SOD, and TRx1 in peripheral blood [127]. This may be crucial for the maintenance of redox control, and may trigger physiological adaptation during strenuous and exhaustive physical exercise, which may impair Trx1 homeostasis and lead to RS [128].

## 7. Adverse Effects of Antioxidant Agents

On the other hand, the protective effect of some compounds having an antioxidant effect is well known. The intra or extracellular antioxidant defenses can scavenge several radicals, eliminate proteins damaged by free radicals, suppress oxidized fatty acids from membranes, and undo damage to DNA caused by free radicals. However, the use of antioxidants is not completely effective for treating neurodegenerative diseases, chronic inflammation, cardiovascular diseases, and cancer, and can even increase the production of free radicals. High doses of antioxidants can also lead to cellular dysfunction, by altering the redox balance after interacting with physiological concentrations of ROS [113]. Thereby, antioxidants may increase the damage to the body by interfering with the metabolism of some nutrients, increase the risk of cancer, or reduce the effectiveness of cancer treatments (e.g., radiation therapy, chemotherapy), thus decreasing the health-promoting effects of exercise, and even decreasing life expectancy [129]. The next section addresses the side effects of some chemical compounds that are used as antioxidants. The side effects of several agents are summarized in Table 2.

### 7.1. Tocopherol

Tocopherol (vitamin E) is the main chain-breaking antioxidant soluble in lipids, plasma, and red cells. It has beneficial antioxidant effects [130]. The rate of tocopherol decay is α > β > γ > δ, in analogy to the biological potencies of these forms of vitamin E [131]. However, only a few articles have shown the effect of high concentrations or chronic consumption of vitamin E supplements. Bone mass and architecture in male rats is altered by the chronic consumption of high levels of dietary vitamin E [132,133]. There is a positive association between increased hs-CRP levels and a high-dose of ingested vitamin E (400 IU/day or more). Mortality by all causes in women is elevated by supplements, and this may be due the pro-oxidant effects of vitamin E [134]. α-Tocopherol in high concentrations acts as a pro-oxidant in in vitro systems, depending on the presence of transition metals [132]. In addition, the use of vitamin E supplement was related with an increased risk of lung cancer, especially in the risk of lung adenocarcinoma [133]. These experimental studies showed that high amounts of α-tocopherol can induce apoptosis. A prospective cohort study assessed the daily use of supplemental vitamin E in women and men aged 50–76 years over 10 years. The supplementation led to a small increase in lung cancer risk. This risk of supplemental vitamin E was mostly shown in smokers, and was at the greatest level for non-small cell type of lung cancer [135]. There was a 7% increase in the risk for each 100 mg/day, and therefore, the increased risk for lung cancer was 28% when ingesting 400 mg/day of vitamin E for 10 years [136,137]. In the Shanghai Women’s Health Study, there was an inverse association in women receiving 14 mg/day (adequate intake of tocopherol) or more with the risk of lung cancer, when compared to those receiving a lower dose [136].

### 7.2. β-Carotene

β-Carotene is a chemical compound of the family of terpenes; β-carotene is the most abundant carotenoid in nature, and it is the most important pro-vitamin in the human diet [154]. The mucosa of the small intestine transforms it into vitamin A, and it is then stored in the liver as a retinol ester [155]. As a lipo-soluble antioxidant, it reduces the chances of heart attacks and increases the efficiency of the immune system [155]. Low β-carotene consumption rates enhance systemic OS in MS patients [156]. However, β-carotene at high doses can be pro-oxidant, and increase the synthesis and release of TNF-α and interleukin-8, that are pro-inflammatory mediators [157]. β-Carotene and α-retinol (30 mg/day) can induce an increase in the incidence of lung cancer in smokers [140]. β-Carotene increases the risk of cancer when administered as an isolated supplement [141]. Combination of reduced fat and wheat bran decreased the recurrence of large adenomatous polyps, and β-carotene increased the risk of polyp recurrence in women [142].

### 7.3. Ascorbic Acid

Ascorbic acid, also known as vitamin C, is a water-soluble vitamin that is eliminated by the kidney via filtration and active tubular reabsorption, and is metabolized to oxalate. [158]. The ascorbic activity of vitamin C lies in its role as an essential cofactor in hydroxylation reactions involved in the biosynthesis of stable cross-linked collagen. Ascorbic acid scavenges O_2_^−^, H_2_O_2_, OH•, HOCl, and aqueous peroxyl radicals [159]. Ascorbic acid undergoes two-electron oxidation to dehydroascorbic acid, with intermediate formation of the relatively unreactive ascorbyl radical during its antioxidant action [160]. Excess consumption of large amounts of vitamin C does not pose a problem to the general population, because it is disposed of by the kidneys. However, patients on hemodialysis can develop secondary oxalosis [161]. Large amounts of oxalate accumulation result in secondary oxalosis caused by an elevated ingestion, high production, or diminished excretion [162]. Calcium oxalate deposition in the kidneys and high levels of serum and urinary oxalate can be caused by the ingestion of elevated doses of vitamin C [143]. Ascorbic acid plays an important part in the protection of plasma lipids against peroxidative damage caused by several kinds of oxidants [160]. However, in high concentrations, it can act as a pro-oxidant agent, and can produce damage by stimulating LPO [158]. This can be the reason why ascorbic acid is employed as a pro-oxidant in peroxidative reactions involving transition metals, particularly iron and copper by the Fenton reaction [163]. It greatly enhances autoxidation, which is accompanied by the production of O_2_^−^ and H_2_O_2_ [158]. The level of LPO indicates a balance between pro-oxidant and antioxidant activity of ascorbic acid, and may ultimately depend on the status of α-tocopherol [135]. In addition, ascorbic acid reductively decomposes *tert*-butyl hydroperoxide, which can then initiate LPO [164]. In another study in photosensitized red cell membranes, ascorbic acid enhanced LPO [165]. Supplementing the diets in healthy individuals with high doses of vitamin C (500 mg/day) produced an elevation in oxidative damage to lymphocyte DNA, suggesting pro-oxidative effects at elevated doses [144].

### 7.4. N-Acetylcysteine

*N*-Acetylcysteine (NAC) is a drug with mucolytic properties that also has antioxidant effects, and is used in the formation of GSH [166]. Chronic treatment with 1 mM NAC on L6 myoblasts induced cellular RS that impaired mitochondrial function of myoblasts and cardiomyocytes by the reduction of the NAD^+^/NADH ratio and Trx2 [1,17,54]. NAC (0.4 mM) induced a reduction–oxidation of the redox state of mitochondria [17]. In human embryonic kidney 293 T cells, NAC treatment resulted in overexpression of the catalytic subunit, GCL, or modified the GCL subunit, favoring a GSH increase, and causing mitochondrial oxidation and cytotoxicity. Thus, it caused an excess GSH that led to RS [17]. Additionally, NAC and vitamin E, or the combination of both, markedly increased tumor progression and reduced survival in mice and human subjects having B-RAF and K-RAS-induced lung cancer [138]. In another study, an association between NAC, ROS reduction, and p53 expression was found. p53 is a major tumor suppressor that acts as a suppressor of inflammation. The inactivation of p53 increases tumor growth by disrupting the ROS–p53 axis. This has consequences in early tumors or precancerous lesions in patients that smoke, and in patients having chronic obstructive pulmonary disease [139].

### 7.5. Synthetic Antioxidants

Several studies have suggested the potential adverse effects of synthetic antioxidants, such as butylated hydroxyanisole (BHA) and butylated hydroxytoluene (BHT) in rodents [145] and monkeys [146]; carcinogenic effects and toxicity were found at high doses. Spoilage in food items, instead of a prolongation of shelf-life, has also been found to increase with high concentrations of synthetic antioxidants, such as BHT and BHA, due to their pro-oxidant activities [146]. BHT has become a model to study lung toxicity; it is being used as a tool in animals, in which it mimics respiratory distress and interstitial pulmonary fibrosis [167]. However, BHT and BHA can induce hypertrophy in the liver of various animal species, including rats, mice, dogs, pigs, and monkeys [168]. In rats, oral administration of a high dose of BHT leads to centrilobular necrosis, accompanied by initial GSH depletion [168]. Injury by BHT in the kidney has also been described [169]. Also, a P450-derived metabolite of BHT (BHT-BuOH) is a more potent tumor promoter in mouse lung, than is BHT [170]. Likewise, feeding subjects with high doses of BHA may lead to the formation of papillomas and squamous cell carcinomas in the fore stomach of rats, hamsters, and mice [171]. Another study showed that feeding BHA at 2% in the diet for nearly the whole lifetime resulted in malignancies in rats [172]. In animals, synergism between BHA and BHT has caused aggravation of pulmonary toxicity [147]. BHA directly inhibits the activities of CYP17A1 and HSD3B1, and the levels of expression of Hsd17b3 and Srd5a1, resulting in diminished androgen production in Leydig cells [148].

### 7.6. Phenolic Antioxidants

Food constituents of plants, such as polyphenols, have cyto-protective activity and preventive effects against OS in vitro; however, they can also display pro-oxidant activities when consumed at elevated doses or when metal ions are present [173]; the concentration determines their pro-oxidant and/or antioxidant activity. Pro-oxidative activities of several polyphenols, such as quercetin, catechins, and gallic acid, have been reported in recent studies that used cell models [174]. Cell survival and viability, thiol content, total antioxidant capacity, and SOD, CAT, and GST activities were reduced at quercetin concentrations of 50 μM [175]. Elevated levels of flavonoids (50–250 μM) resulted in cytotoxicity, damage to DNA, apoptosis, and presence of ROS by autoxidation [149]. Phenolic antioxidants at high concentrations display pro-oxidant activities when transition metal ions such as iron and copper are present, forming chelators and reducing the antioxidant capacity [176]. Phenolic antioxidants are converted into phenoxyl radicals. In biological systems, phenoxyl radicals can be the basis of a cascade of pro-oxidative events which are characterized first by autoxidation of a diphenol or polyphenol, concomitant with a univalent reduction of molecular oxygen, followed by dismutation of the O_2_^−^ formed, and subsequent formation of hydroxyl radicals in a Fenton-type reaction [177]. These diphenolic compounds are more cytotoxic than monophenolic substances because they produce much larger quantities of reactive oxygen metabolites in the extracellular space [175]. Quercetin is a flavonoid that may lead to H_2_O_2_ formation during autoxidation [178]. Excess production of H_2_O_2_ in microsomes has been observed with a number of phenolic antioxidants, such as quercetin and gallates [179]. Regarding the pro-oxidant effect of phenolic agents, it was recently reported that an infusion of 3% of Hibiscus sabdariffa L (HSL), a plant that possesses a large amount of polyphenols, reduces the pathologies that comprise MS, including hypertension hyperinsulinemia, IR, obesity, and OS in a rat model, caused by administration of 30% sucrose in the drinking water. The reduction of OS was due to an increase of SOD, CAT, and GPx, and decrease of hypertension, LPO, and carbonylation [180]. However, infusion at 6% in drinking water in this model overexpresses the antioxidant enzymes, and might result in an increase in blood pressure and probably RS. However, more studies are needed to confirm this observation. Resveratrol, a naturally occurring antioxidant present in red wine, exerts cardiovascular protection by reducing OS and non-esterified fatty acid [181]. In nM concentrations, resveratrol can enhance endothelial NO production through a caveolae-dependent mechanism involving p42/44^MAPK^ activation [150]. However, 10–25 μM resveratrol can also induce pro-oxidant effects in a dose-dependent pattern, provoking mitochondrial damage and endothelial cell death through CYP2C9 [150], by down-modulating Akt phosphorylation [182]. In a similar way to other natural antioxidants, such as coumaric, chlorogenic, ferulic, caffeic, and caftauric acids, food-derived phenolic compounds at a high-dose (25 μM) can increase intracellular ROS production and have pro-oxidant effects through the flavin-containing CYP450 families [183]. Coumaric acid, a common dietary polyphenolic antioxidant, can also induce intracellular pro-oxidant effects in human endothelial cells and death mediated by CYP2C9 [151].

### 7.7. Estrogens

The antioxidant action of estrogens, and especially of 17β-estradiol, is displayed by two mechanisms; the first is through its hydroxyphenolic structure, that may donate hydrogen atoms resulting in the capture of ROS and cell membrane LPO [153]. The second mechanism is associated with its stimulatory effect on cellular antioxidant enzyme genes [184]. However, estrogens at high concentration may induce damage to the cell by OS development through metabolic reactions of the phenolic ring, which becomes its predominant biochemical activity and could exert deleterious effects. The oxidations of estrogens to catechol estrogens, and further to quinones, induce ROS by redox cycling of estrogens [185]. The quinones formed from catechol estrogens are considered pro-oxidants due to the production of ROS through redox cycling via semiquinones [186]. Estrogens metabolized to phenoxyl radicals, quinones or semi-quinones, may cause damage in cells either through alkylation or oxidation of cellular macromolecules, including DNA [152]. Estrogens are hydroxylated by NADPH-dependent cytochrome P450 enzymes to catechol estrogens, and consume O_2_, inducing DNA strand break. Through their capacity to donate electrons, they promote neoplastic transformation and the development of breast cancer [170].

## 8. Summary and Conclusions

In summary, RS is characterized by an excess of reducing equivalents. It leads to a decrease of ROS production through antioxidant enzyme overexpression that may cause an alteration in the redox state of intracellular higher NAD^+^/NADPH, and GSH/GSSG ratio. A balance in Se and iron levels is needed for several biological functions in the human body, and its excess and/or insufficient intake can result in adverse health effects and contribute to RS. RS alters the mitochondrial function, causes misfolding of proteins, and may participate in several inflammation-associated diseases. Hyperglycemic conditions induce RS through inhibition of the insulin receptor by selenium-GPx-1 overexpression. Antioxidant vitamins, polyphenols and estrogens ingested in high concentrations can induce a pro-oxidant state with adverse effects for the organisms.

In conclusion, recent information shows the importance of the redox regulation for cellular homeostasis. Excess ROS (oxidative stress) or of reducing equivalents (reductive stress) alter the regulation of cellular signaling pathways, leading to several diseases. There are many sources of RS, and its generation alters different cellular processes, such as mitochondrial function, transcription, translation, and post-translational modifications. An elevated ingestion of supposedly “healthy” compounds, such as antioxidant vitamins, synthetic antioxidants, polyphenols, or hormones (estrogens), can induce a pro-oxidant state, which generates RS with adverse effects for the organism.

## Figures and Tables

**Table 1:** Inflammation-associated diseases linked to reductive stress.

Disease	Source of RS	References
Cardiomyopathy	↑ GSH/GSSG ratio	Rajasekaran et al., 2007 [60]; Bauersachs et al., 2010 [61]; Brewer et al., 2013 [59]; Baek et al., 2000 [62]; Rajasekaran et al., 2011 [63]
	↓ Free iron content	Zhang et al., 2010 [64]
Pulmonary hypertension	↑ NADPH/NADP^+^ ratio	Oldham et al., 2015 [65]
Stent stenosis	↑ GSH/GSSG ratio ↑ NADPH/NADP^+^ ratio	de Haan., 2014 [66]
Muscular dystrophy	↑ GSH/GSSG ratio	Rajasekaran et al., 2007 [60]; Dialynas et al., 2015 [58]
Neurological disorders	↑ Selenium levels	Tsunoda et al., 2000 [67]; Ayaz et al., 2008 [68]
	↑ GPx activity	Ince et al., 1994 [69]
Parkinson’s disease	↑ NADH/NAD^+^ ratio	Greenamyre et al., 2010 [70]
Alzheimer’s disease	↑ G6PD and GSH	Lloret et al., 2016 [71]; Russell et al., 1999 [72]
Metabolic syndrome and insulin resistance	↑ GPx1 expression ↑ NADPH/NADP^+^ ratio	McClung et al., 2004 [73]
Rheumatoid arthritis	↑ NADPH/NADP^+^ ratio	Yang et al., 2016 [74]
Renal diseases	↑ GSH/GSSG ratio	Li et al., 1993 [75]
	↑ Thiols	Welch et al., 1992 [76]
Cancer	↑ NADH/NAD^+^ ratio	Oldham et al., 2015 [66]

(↓): reduction; (↑): increase. Abbreviations: RS: reductive stress; GSH: glutathione; GSSG: glutathione disulfide; G6PD: glucose-6-phosphate dehydrogenase; NADH: Nicotinamide adenine dinucleotide reduced; NAD^+^: Nicotinamide adenine dinucleotide oxidized; NADPH: Nicotinamide adenine dinucleotide phosphate reduced; NADP^+^: Nicotinamide adenine dinucleotide phosphate oxidized; GPx: gluthathione peroxidase 1.

**Table 2:** Side effects of antioxidant agents in inflammation-associated diseases.

Antioxidant Agent	Mechanisms	Associated Pathology	References
Tocopherol or Vitamin E	Pro-oxidant activity by Fenton reaction	Bone alterations lung cancer	Smith et al., 2005 [132]; Iwaniec et al., 2013 [133]; Wu et al., 2015 [136]; Slatore et al., 2008 [137]
NAC	Reduction of NAD^+^/NADH ratio	Cardiovascular disorders Lung cancer	Zhang et al., 2012 [17]; Mendelsohn et al., 2014 [138]; Sayin et al., 2014 [139]
β-carotene	Pro-oxidant and pro-inflammatory	Cancer Colorectal polyps	Goodman et al., 1996 [140]; Bjelakovic et al., 2015 [141] MacLennan et al., 1995 [142]
Ascorbic acid (Vitamin C)	Pro-oxidant activity by Fenton reaction	Renal calcium oxalate deposition DNA damage of lymphocytes	Hatch et al., 1980 [143]; Podmore et al., 1998 [144]
BHA and BHT	Pro-oxidative properties	Cancer Pulmonary toxicity Reproductive damage	Branen, 1975 [145]; Ito et al., 1983 [146]; Thompson et al., 1989 [147] Li et al., 2016 [148]
Flavonoids	Pro-oxidant activity by Fenton reaction	DNA damage, Apoptosis Hypertension	Hodnick et al., 1986 [149]
Resveratrol	Pro-oxidant by CYP2C9	Endothelial cell death	Posadino et al., 2015 [150]
Coumaric Acid	Pro-oxidant Mitochondrial Damage	Endothelial cell death	Posadino et al., 2013 [151]
Estrogens	Pro-oxidative properties	Cell damage Breast cancer	Ayres et al., 1998 [152] Bednarek, 2002 [153]

Abbreviations: NAC: *N*-acetylcysteine; NADH: Nicotinamide adenine dinucleotide reduced; NAD^+^: Nicotinamide adenine dinucleotide oxidized; BHA: Hydroxyanisole; BHT: butylated hydroxytoluene.
